# Pathogenic variants at the N-terminal arginine residue 44 disrupt human GABA transporter 1 function: insights from *Drosophila* epilepsy models

**DOI:** 10.3389/fphar.2025.1674737

**Published:** 2025-11-25

**Authors:** Nikita Shah, Ameya Sanjay Kasture, Vasylyna Kovalchuk, Lara Bjeletic, Thomas Hummel, Harald H. Sitte, Sonja Sucic

**Affiliations:** 1 Institute of Pharmacology, Center of Physiology and Pharmacology, Medical University of Vienna, Vienna, Austria; 2 Department of Neuroscience and Developmental Biology, University of Vienna, Vienna, Austria; 3 Imperial College London, London, United Kingdom; 4 Hourani Center for Applied Scientific Research, Al-Ahliyya Amman University, Amman, Jordan; 5 Center for Addiction Research and Science, Medical University of Vienna, Vienna, Austria

**Keywords:** γ-aminobutyric acid (GABA) transporter 1 (GAT-1), *Drosophila melanogaster*, epilepsy, proteasome inhibition, small molecules, disease variants

## Abstract

Arginine 44 (R44) is a highly conserved residue in the human GABA transporter 1 (hGAT-1), a member of the solute carrier 6 (SLC6) family, which plays a critical role in regulating inhibitory neurotransmission in the central nervous system. To elucidate its functional importance, we characterized three epilepsy-associated pathogenic variants - R44Q, R44P and R44W - linked to myoclonic-atonic epilepsy (MAE) and developmental delay. Building on evidence that R44 resides within the N-terminal intracellular gate and is essential for transporter function, we employed biochemical, cellular and organismal models (HEK293 cells and *Drosophila melanogaster,* respectively) to assess the variants’ functional impairments, subcellular localization and trafficking. Particular emphasis was placed on R44Q, a validated variant with a pronounced clinical phenotype. The mutants trafficked to the plasma membrane, but were non-functional and exhibited reduced protein stability. *In vivo*, R44Q displayed cell-type–specific degradation: in astrocytes, GAT was rapidly cleared via proteasomal degradation, whereas in neurons, it showed lower expression with presynaptic enrichment. Proteasome inhibitors (MG-132, bortezomib) and the HDAC inhibitor trichostatin A (TSA) partially rescued R44Q function. Moreover, R44Q-expressing flies presented heat-induced seizures, which were mitigated by 4-phenylbutyrate (4-PBA) treatment. These findings elucidate the molecular basis of R44-mediated hGAT-1 dysfunction and highlight potential therapeutic avenues for SLC6A1-related neurodevelopmental disorders.

## 1 Introduction

The primary inhibitory neurotransmitter, γ-aminobutyric acid (GABA), is regulated in the central nervous system by four specific human GABA transporters (hGATs), with hGAT-1 serving as the principal isoform expressed at presynaptic terminals (Kanner, 1978; Guastella et al., 1990; Minelli et al., 1995; Conti et al., 1998). hGAT-1, the first member of the solute carrier 6 (SLC6) family (*SLC6A1*), plays a critical role in GABA uptake from the synaptic cleft into neurons and astrocytes, thereby regulating inhibitory neurotransmission (Borden, 1996). The human GAT-1, comprising 599 amino acids, contains 12 transmembrane domains (TMDs). Both its amino (N) and carboxyl (C) termini are located intracellularly. A large extracellular loop between transmembrane helices 3 and four harbors three consensus sites for N-linked glycosylation (Bennett and Kanner, 1997). Kinetic and thermodynamic studies have shown that GABA translocation via GAT-1 is electrogenic, relying on an inward electrochemical gradient and the co-transport of Na^+^ and Cl^−^ ions across the synaptic membrane. Binding of Cl^−^ to GAT-1 on the extracellular side is thought to facilitate the intracellular release of GABA from the transporter (Zomot et al., 2007; Scimemi, 2014). In this process, the outward-open conformation permits the binding of substrate and co-transported ions, which triggers closure of the extracellular gate, forming an occluded conformation that prevents substrate and ion dissociation. This is followed by the opening of the intracellular gate in the inward-open conformation, allowing the release of GABA into the cytoplasm (Motiwala et al., 2022; Kanner and Dayan-Alon, 2023).

At the physiological level, GABAergic signaling - mediated through the activation of downstream ionotropic and metabotropic GABA receptors - is finely modulated by the precise timing of GABA release from presynaptic terminals into the extracellular space and its subsequent clearance from the synaptic cleft. Impairments in this tightly regulated process can lead to dysfunctional GABAergic transmission, which is associated with various neurological disorders. Pathogenic mutations in hGAT-1 were first identified in patients with myoclonic-atonic epilepsy (MAE), a generalized epilepsy syndrome characterized by multiple seizure types - including myoclonic, myoclonic-atonic, atonic, and absence seizures - each exhibiting variable severity and clinical presentation (Mattison et al., 2018). Beyond epilepsy, individuals with MAE (also known as Doose syndrome) often present with a broader neurodevelopmental phenotype, ranging from mild to severe intellectual disability and developmental delay, frequently accompanied by features of autism spectrum disorder (reviewed in (Fischer et al., 2022; Shah et al., 2024). In this study we focused on three disease variants at a highly conserved arginine residue 44 (R44), located within the N-terminal domain of hGAT-1 (Table 1). Prior studies have demonstrated the significant role of the N-terminus of rat GAT-1 in transport activity, specifically highlighting the segment spanning residues 42 to 49, while residues 3 to 42 were found to have no major impact on GAT-1 function (Bennett et al., 2000). Residue R44, located near the cytoplasmic surface, forms a key interaction with aspartate 410 (D410), which is assumed to act as a narrow intracellular gate in the GABA transporter. This interaction likely contributes to the closure of the internal gate, enabling the transporter to reorient into its outward-facing conformation. (Ben-Yona and Kanner, 2013). Synthetic R44 mutations (to serine, histidine and lysine) displayed marked functional defects, likely induced by impeding the GABA translocation cycle (Bennett et al., 2000). Mutations in the equivalent charged residue pair (R60 and D436) of the dopamine transporter (DAT) disrupted the salt bridge, resulting in an increased proportion of transporters adopting the inward-facing conformation (Kniazeff et al., 2008). This, in turn, highlights the interdependence and interactions of these intracellular gate residues and their role in mutually stabilizing the transporter conformation.

**TABLE 1:** Sequence alignment of SLC6 transporters highlighting the conserved arginine residue.

Transporter	Gene	Name	Uniprot ID	Partial sequence
Human SLC6 proteins	*slc6a1*	GAT1	P30531	VKVQKKAADLPD**R** ^**44**^DTWKGRFDFLMS
	*slc6a2*	NET	P23975	---PR—DGDAQP**R** ^**56**^ETWGKKIDFLLS
	*slc6a3*	DAT	Q01959	TNPRQSPVEAQD**R** ^**60**^ETWGKKIDFLLS
	*slc6a4*	SERT	P31645	TTTLVAELHQGE**R** ^**79**^ETWGKKVDFLLS
	*slc6a5*	GlyT2	Q9Y345	TQEDEQGDENKA**R** ^**191**^GNWSSKLDFILS
	*slc6a6*	TAUT	P31641	RPEDEAEGKPPQ**R** ^**41**^EKWSSKIDFVLS
	*slc6a7*	PROT	Q99884	GDVDLDVDFAAH**R** ^**37**^GNWTGKLDFLLS
	*slc6a8*	CRT1	P48029	LGTPGGRLAVPP**R** ^**52**^ETWTRQMDFIMS
	*slc6a9*	GlyT1	P48067	PSEATKRDQNLK**R** ^**100**^GNWGNQIEFVLT
	*slc6a11*	GAT3	P48066	HPRVKRDKAVHE**R** ^**50**^GHWNNKVEFVLS
	*slc6a12*	BGT1	P48065	KLDQEDEDQVKD**R** ^**36**^GQWTNKMEFVLS
	*slc6a13*	GAT2	Q9NSD5	VMEKKEEDGTLE**R** ^**32**^GHWNNKMEFVLS
	*slc6a14*	ATB^0+^	Q9UN76	NFHVGENDENQD**R** ^**36**^GNWSKKSDYLLS
	*slc6a15*	B^0^AT2	Q9H2J7	TDVEEGSEVEDE**R** ^**61**^PAWNSKLQYILA
	*slc6a16*	NTT5	Q9GZN6	MTEKKESEVLLA**R** ^**103**^PFWSSKTEYILA
	*slc6a17*	NTT4	Q9H1V8	QKAVEEELDAED**R** ^**60**^PAWNSKLQYILA
	*slc6a18*	B^0^AT3	Q96N87	EPDPAACDLGDE**R** ^**18**^PKWDNKAQYLLS
	*slc6a19*	B^0^AT1	Q695T7	ELETIEQEEASS**R** ^**32**^PKWDNKAQYMLT
	*slc6a20*	IMINO	Q9NP91	--------MEKA**R** ^**5**^PLWANSLQFVFA
GABA transporters				
*C. elegans*	*snf3*	SNF3	G5EBN9	ELEQSQHSEEPD**R** ^**30**^GQWTGKFDFLMS
Zebrafish	*slc6a1b*	GAT1b	Q5U3E5	VKVQKK-KELPD**R** ^**43**^ETWQGKFDFLLS
*Drosophila*	*gat*	GAT	Q9V4E7	KQQLVKIEQLPD**R** ^**58**^GSWSSKMDFILS
Rat	*slc6a1*	GAT1	P23978	VKVQKKAADLPD**R** ^**44**^DTWKGRFDFLMS
Rat	*slc6a13*	GAT2	P31646	-MEKVEEDGTLE**R** ^**32**^EQWTNKMEFVLS
Rat	*slc6a11*	GAT3	P31647	-TREARDKAVHE**R** ^**45**^GHWNNKVEFVLS
Rat	*slc6a12*	BGT1	P48056	-MDQKDKDQVKD**R** ^**36**^GQWTNKMEFVLS
Mouse	*slc6a1*	GAT1	P31648	VKVQKKAADLPD**R** ^**44**^DTWKGRFDFLMS
Mouse	*slc6a13*	GAT2	P31649	-MEKVEEDGTLE**R** ^**32**^EQWTNKMEFVLS
Mouse	*slc6a11*	GAT3	P31650	-TREARDKAVHE**R** ^**45**^GHWNNKVEFVLS
Mouse	*slc6a12*	BGT1	P31651	-MDQKDKDQVKD**R** ^**36**^GQWTNKMEFVLS
Bovine	*slc6a1*	GAT1	A0AAF6DMF8	VKVQKKAADLPD**R** ^**44**^DTWKGRFDFLMS
Bovine	*slc6a6*	TAUT	Q9MZ34	RPEDEAEGKPPQ**R** ^**41**^EKWASRIDFVLS
Bovine	*slc6a13*	GAT2	A5PJX7	-LEKAAEDGALQ**R** ^**32**^EQWSNKMEFLLS
Other transporters				
Aquifex aeolicus (strain VF5)	*snf*	Na^+^: Neurotransmitter symporter	O67854	--------MEVK**R** ^**5**^EHWATRLGLILA

A multiple sequence alignment of SLC6 transporters, showing the highly conserved arginine (R) residue (indicated in bold font), corresponding to position 44 in the human GAT-1 (*slc6a1*).

Here, we unravelled the molecular pathogenesis of epilepsy-associated hGAT-1 mutations, R44Q, R44P and R44W. The arginine-to-glutamine substitution at position 44 (R44Q; ClinVar RCV001092965) was clinically reported in an 8-year-old patient with myoclonic-atonic epilepsy (MAE) and developmental delay, with seizure onset at 2.5 years of age and no known family history (Carvill et al., 2015). Additional missense variants at the same residue - arginine-to-proline (R44P; ClinVar RCV003596534) and arginine-to-tryptophan (R44W; ClinVar RCV000524089) - have also been identified in individuals with MAE, although the precise molecular mechanisms by which these mutations contribute to disease remain undefined. To elucidate the functional consequences of these variants, we employed a combination of pharmacological, biochemical assays and imaging approaches to probe transporter activity and subcellular localization in both *in vitro* (HEK293 cells) and *in vivo* (*Drosophila melanogaster*) models. Given the relatively non-conservative nature of the R44Q substitution (i.e., eliminating a positive charge and appreciably reducing side-chain length) and its association with a severe clinical phenotype, we prioritized this variant for detailed mechanistic analysis and therapeutic targeting. Our findings highlight the indispensable role of the N-terminal domain in maintaining hGAT-1 structural stability and activity, and implicate its disruption as a major contributor to epileptogenesis in these patients.

## 2 Materials and methods

### 2.1 Reagents

[^3^H]GABA (specific activity: 25–40 Ci/mmol; cat. no. NET191250UC) was purchased from Revvity Inc. (Boston, USA). Dulbecco’s Modified Eagle Medium (DMEM), supplements and other cell culture reagents were obtained from Invitrogen. 17-Dimethylaminoethylamino-17-demethoxygeldanamyci (17-DMAG; cat. no. CAY11036-5) was from Cayman Chemical (Michigan, USA). Pifithrin-µ (cat. no. 506132), 4-phenyl butyric acid (4-PBA; cat. no. P21005-25G), tiagabine (cat. no. SML0035-10 MG) and 0.4% trypan blue solution (cat. no. T8154-100 ML) were all purchased from Sigma–Aldrich (St. Louis, MO, USA). Other reagents, such as Complete™ protease inhibitor cocktail and sodium dodecyl sulfate (SDS) were from Roche Applied Science (Mannheim, Germany) and BioMol GmbH (Hamburg, Germany; cat. no. 51430), respectively. Bortezomib and MG-132 were purchased from TargetMol Chemicals Inc. (Boston, MA; cat. no. T2399-1 mL) and Sigma-Aldrich (cat. no. 474790-1 MG), respectively. Trichostatin A was from Sigma-Aldrich (cat. no. T1952-200UL). Endoglycosidase H was obtained from New England Biolabs GmbH (MA, USA; cat. no. P0702S). Tris and scintillation liquid (Rotiszint® eco plus) were provided by Carl Roth GmbH (Karlsruhe, Germany). All other commercial solvents and common use chemicals were of analytical grade. Primary antibodies, rabbit polyclonal anti-green fluorescent protein (GFP; cat. no. ab290) and mouse monoclonal anti mCherry (cat. no. ab125096) were acquired from Abcam Plc (Cambridge, UK). Secondary antibodies, goat anti-rabbit IgG (cat. no. 926-68071) IRDye® 680RD and donkey anti-mouse IgG (cat. no. 926-32212) IRDye® 800CW were from LI-COR (Lincoln, Nebraska, USA) and used at a dilution of 1:5000.

### 2.2 Site-directed mutagenesis and cloning

The desired hGAT-1 mutations were generated using the QuikChange Lightning site-directed mutagenesis kit (Agilent Technologies, Santa Clara, CA; cat. no. cat. no. 210518), with the WT hGAT-1 (OriGene, cat. no. RC206290) subcloned into the pEYFP-C1 vector (Addgene; cat. no. 6006-1) (YFP-hGAT-1) used as template. Mutagenic primers were designed using the QuikChange primer design tool, and were: ctt​cca​cgt​gtc​ctg​gtc​ggg​gag​gtc for R44Q, tcc​acg​tgt​ccg​ggt​cgg​gga​gg for R44P and tcc​acg​tgt​ccc​agt​cgg​gga​ggt​c for R44W (sequences shown are forward strands, in the 5′to 3′direction). The mutations were confirmed by automatic DNA sequencing (LGC Labor GmbH Augsburg, Germany).

### 2.3 Cell culture, transient transfections and drug treatments

Human embryonic kidney (HEK293; American Type Culture Collection (ATCC); cat. code CRL-1573) cells were cultured in complete Dulbecco’s modified Eagle’s medium (DMEM) containing high glucose (4.5 g/L) and L-glutamine (584 mg/L), with 10% fetal bovine serum and penicillin (60 mg/L) and streptomycin (100 mg/L). The cells were kept in a humidified atmosphere (37 °C and 5% CO_2_). Post 24 h of seeding cells, they were transiently transfected with plasmids encoding wild-type YFP-hGAT-1 or the mutants of interest, using Lipofectamine 2000 (Life Technologies, Carlsbad, CA) or polyethylenimine (PEI) (23,966, Polysciences Europe GmbH, Germany) for 24 h, according to the manufacturer’s protocols. For co-expression experiments, wild-type mCherry-hGAT-1 was co-transfected with YFP-tagged R44Q epilepsy variant at 1:1 ratio (plasmid ratio by weight), keeping the amount of WT DNA constant. For [^3^H]GABA uptake experiments, transiently transfected HEK293 cells were seeded (∼10^5^ cells per well) onto poly-D-lysine (PDL)-coated 48-well plates. Cells were treated with diverse compounds (pharmacochaperones, proteasome inhibitors and trichostatin A) and incubated for an additional 24 h, preceding the functional uptake and biochemical assays. For thermal shift assays, transfected cells expressing the constructs of interest, grown in 10 cm culture dishes, were treated with the proteasome inhibitor MG-132 for 24 h and processed further, as described under ‘Enzymatic de-glycosylation and immunoblotting’.

### 2.4 Radioligand GABA uptake assays

For radiolabeled uptake assays, the media was removed and cells were gently washed with 500 µL Krebs-HEPES buffer (KRH) (10 mM HEPES, 120 mM NaCl, 3 mM KCl, 2 mM CaCl_2_, 2 mM MgCl_2_, 2 mM glucose monohydrate, pH 7.3). The cells were incubated with 50 nM [^3^H]GABA at room temperature for exactly 3 min. For saturation uptake kinetics, [^3^H]GABA was diluted with increasing concentrations of unlabeled GABA spanning from 1 to 30 µM. The cells were next rapidly washed with 500 µL ice-cold KRH buffer to stop the uptake process, thereafter the cells were lysed with 500 µL of 1% SDS solution. Non-specific uptake (∼10% of total uptake) was determined in the presence of tiagabine (10 μM). [^3^H]GABA content was determined by liquid scintillation counting.

### 2.5 Enzymatic de-glycosylation and immunoblotting

48 h post transfection, the cells were washed twice with ice cold phosphate buffered saline (PBS) and further lysed in a NP-40 buffer, containing 50 mM Tris.HCl (pH 7.4), 150 mM NaCl, 1% Nonidet P-40 (NP-40), 10 mM EDTA, supplemented with a protease inhibitor cocktail (Roche Complete™; cat. no. 11836170001). The lysates were incubated by rotation at 4 °C for 1 h. Next, centrifugation (40 min at 16,000 g at 4 °C) was performed to remove debris and insoluble material. 20–25 μg of total protein was incubated in the absence or presence of a de-glycosylation enzyme, endoglycosidase H (EndoH), using the NEB kit (New England Biolabs, MA, USA; cat. no. P0702S), for 2 h at 37 °C, following the instructions of the manufacturer. Equal amounts of protein were added to the SDS denaturing polyacrylamide gel, separated by electrophoresis (10% resolving gel) and electrotransfer was performed onto polyvinylidene difluoride (PVDF) membranes. The membranes were blocked with blocking solution (5% non-fat skimmed milk in Tris-buffered saline, containing 0.1% Tween 20 (TBST)) for 1 h at room temperature and then probed with a rabbit polyclonal anti-GFP antibody (1:5000 dilution) overnight at 4 °C. For co-expression immunoblotting experiments, mouse monoclonal anti-mCherry antibody was used in addition (1:5000 dilution). An antibody against the G protein β-subunit (1:1000 dilution), was also used to assure equal amount of protein loading in all the lanes of the gel. Next day, after repeated washing steps with TBST, the immunoreactivity was detected by fluorescence on Odyssey CLx (9140, LI-COR, Lincoln, Nebraska USA), by incubating the membranes with a goat anti-rabbit secondary antibody (1:5000 dilution; for GFP detection) (IRDye 680RD, LICOR) and donkey anti-mouse secondary antibody (1:5000 dilution; for mCherry detection) (IRDye 800CW, LICOR). Densitometric band analyses were carried out using ImageJ (NIH).

### 2.6 Confocal microscopy and image acquisition

24 h post transfection, HEK293 cells expressing YFP-tagged transporters were seeded onto PDL-coated 8-well ibidi® glass bottom chambers. Images were acquired using a Zeiss LSM980 inverted live cell imaging microscope, with a ×63 oil objective (NA 1.4). Trypan blue solution (0.05% in PBS) was used to stain the plasma membranes. Acquisition settings were kept constant (pinhole = 1 AU, gain = 700–900). The images were obtained at a resolution of 1024 × 1024.

### 2.7 Thermal stability assays

The lysates, with and without MG-132 exposure, were incubated at room temperature (i.e., non-heated controls) or heated (at increasing temperatures, ranging from 52 °C to 60 °C, for 15 min). The samples were cooled at room temperature (RT) for 5 min and used for immunoblotting, as described in under ‘Enzymatic de-glycosylation and immunoblotting’.

### 2.8 *In silico* analysis

Structural alterations induced by the R44Q mutation were analyzed using ColabFold v1.5.5 software (Mirdita et al., 2022). The generated PDB files were analyzed using ChimeraX 1.9 (Meng et al., 2023). AlphaFold PDB P30531 (GAT-1) served as a control in the structural analyses. The hGAT-1 protein exhibits intrinsically disordered regions in its cytoplasmic amino (N-) and carboxyl (C-) termini. The ColabFold v1.5.5 software predicts per-residue confidence metrics (pLDDT), with low values estimated for cytoplasmic domains. The remaining hGAT-1 structure showed high pLDDT values, except for residues 358 to 363 (https://alphafold.ebi.ac.uk/entry/P30531).

### 2.9 *Drosophila* genetics, immunohistochemistry and imaging

The transgenic reporter line for the wild-type YFP–tagged hGAT-1 was already available from our previous studies (Kasture et al., 2023). The YFP–tagged R44Q-hGAT-1 was subcloned into the pUASg attB vector (Bischof et al., 2007) and microinjected into ZH-86Fb (Bloomington stock no. 24749) fly embryos. The landing site was kept constant to avoid any positional effect. All flies were maintained at 25 °C in a 12-h light/12 h dark cycle, and the crosses were performed at 25 °C. ALRM Gal4 (astrocytic leucine-rich repeat molecule Gal4, Bloomington stock no. 67031) was used drive the expression in astrocytes. GAD-1 Gal4 (Glutamic Acid Decarboxylase 1 Gal4, Bloomington stock no. 51630) was used to drive the expression in GABAergic neurons. SS02766 Gal4 (BDSC 75994) was used to drive the expression in subset of GABAergic ring neurons. UAS-mCD8-GFP (FBti0012686) and UAS-KDEL-RFP (Bloomington stock no. 30909) were used delineate cellular membrane and endoplasmic reticulum, respectively. RPT1 RNAi (Bloomington stock no. 33930) and RPT6 RNAi (Bloomington stock no. 34712) were used to knockdown respective genes using the RNAi pathway. Flies of the said genotype received standard cornmeal medium/food, supplemented with either water, 1 mM 4-PBA, 100 μM tiagabine or 100 μM liothyronine. 4-PBA and tiagabine were dissolved in water (medium) and 50 mM stock solutions of liothyronine were prepared in DMSO. Flies were fed for 48 h and used in subsequent imaging and behavior studies.

Adult fly brains were dissected in PBS and fixed in 4% paraformaldehyde in PBS, for 20 min at room temperature. The brains were washed in 0.1% Triton X-100 in PBS (PBST) three times for 20 min and blocked in 10% goat serum for 1 h at room temperature. They were next incubated in solutions containing antibodies directed against GFP (1:1000 dilution; A-11122, Invitrogen, Vienna, Austria) and neuronal cadherin (1:20, DSHB, Iowa, IA, USA), overnight in 10% goat serum at 4 °C. Next, the brains were washed three times in PBST for 20 min before incubating with Alexa Fluor 488 goat anti-rabbit IgG (1:500; Invitrogen, Vienna, Austria) and Alexa Fluor 647 goat anti-mouse IgG (1:500; Invitrogen, Vienna, Austria) secondary antibodies in 10% goat serum for 3 h at room temperature. The brains were washed three times with PBST and were mounted using Vectashield® (Vector Laboratory, Burlingame, CA, United States). Images were captured on a Leica SP5II confocal microscope with 20-fold magnification. Z-stack images were scanned at 1.5 μm section intervals with a resolution of 512 × 512 pixels. Images were processed in ImageJ. Endogenous dGAT was not visualized by confocal microscopy.

### 2.10 Heat-induced seizure assays in flies

The seizure assay was performed as described earlier (Kasture et al., 2023). Briefly, at least ten 3–5-day-old male flies of said genotypes were immersed in a water-bath at 40 °C for a period of 1–5 min. Mechanical or thermal stimulation results in a distinct repertoire of seizure-like behaviors (Lee and Wu, 2002; Burg and Wu, 2012). Seizure-like episodes were defined as an initial period of uncoordinated movements, brief leg twitches and wing flapping, followed by failure to maintain a standing posture that leads to a paralysis state, in which the fly displays a lack of motion or responsiveness. This is followed by a recovery phase, during which flies attempt to regain posture, manifested by wing flapping and leg twitching. The flies eventually recover normal posture.

### 2.11 Statistical data analysis

All data were analyzed using GraphPad Prism version 10.2.0 (GraphPad Software, San Diego, CA, USA, www.graphpad.com) and the appropriate statistical comparisons (one-way analysis of variance (ANOVA) with Tukey´s *post hoc* tests or paired t-tests), as indicated in figure legends. The results were obtained from at least three independent experiments, with each data set or condition performed in triplicate in all functional assays.

## 3 Results

### 3.1 Functional and molecular assessment of R44-hGAT-1 variants in HEK293 cells

**FIGURE 1:**
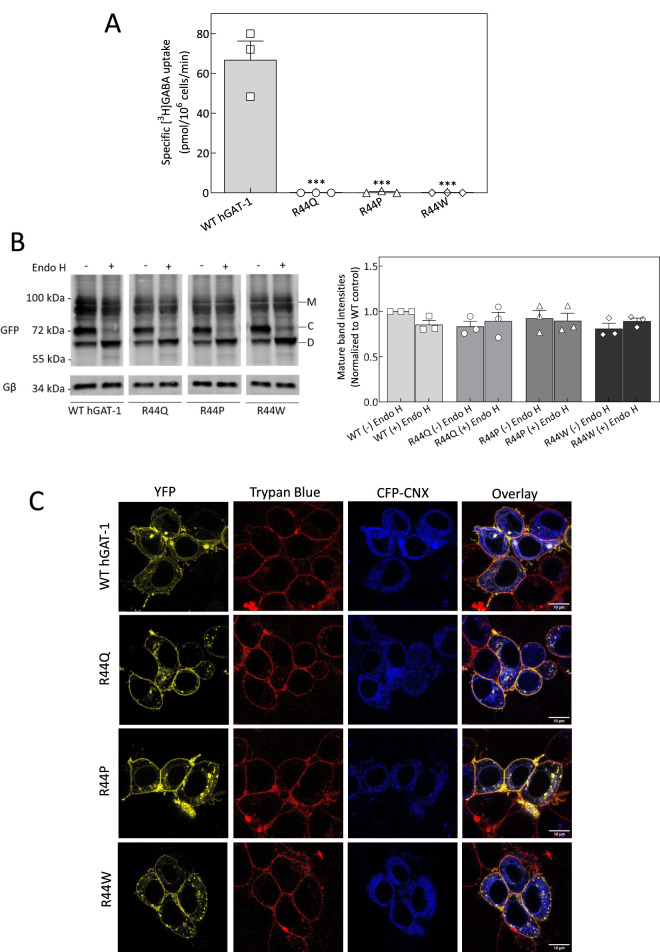
*In vitro* characterization of R44 epilepsy variants of hGAT-1. **(A)** Radioligand [^3^H]GABA uptake assays were conducted in HEK293 cells expressing YFP-tagged GATs to evaluate the transport activity of WT hGAT-1 and the R44 mutants (R44Q, R44P, R44W). Specific GABA uptake was measured over a 3-min period, with non-specific uptake determined in the presence of 10 µM tiagabine. All three R44 mutants had abolished GABA uptake. Data represent the mean ± SEM from at least three independent experiments performed in triplicate. Statistical significance was assessed by one-way ANOVA followed by Tukey’s *post hoc* tests (***, p < 0.001 versus WT). **(B)** Representative immunoblots showing the glycosylation status of WT hGAT-1 and R44 mutants after treatment with endoglycosidase H (Endo H). Bands corresponding to mature glycosylated (“M”), core glycosylated (“C”), and deglycosylated (“D”) forms are indicated. Details of the deglycosylation protocol are provided in the Methods section (left panel). The bands corresponding to the mature glycosylated proteins (“M” bands) were quantified; the intensities were normalized to Gβ and plotted relative to the WT control. The data were obtained from three independent experiments (error bars = SEM). The values were statistically compared by one-way ANOVA, followed by Tukey´s *post hoc* t-tests. There was no significant difference (*p > 0.05) in the surface localization of non-functional mutants R44Q, R44P and R44W, compared to the WT hGAT-1 (right panel). **(C)** Confocal microscopy images of HEK293 cells expressing N-terminal YFP-tagged WT or mutant GATs. Trypan blue staining marks the plasma membrane, while calnexin-CFP labels the endoplasmic reticulum (ER). Scale bars = 10 µm. All mutants exhibited plasma membrane localization comparable to WT, demonstrated by co-localization of YFP fluorescence with trypan blue. The deglycosylation patterns observed in **(B)** align with these imaging results, confirming proper membrane targeting of the R44 mutants.

**FIGURE 2:**
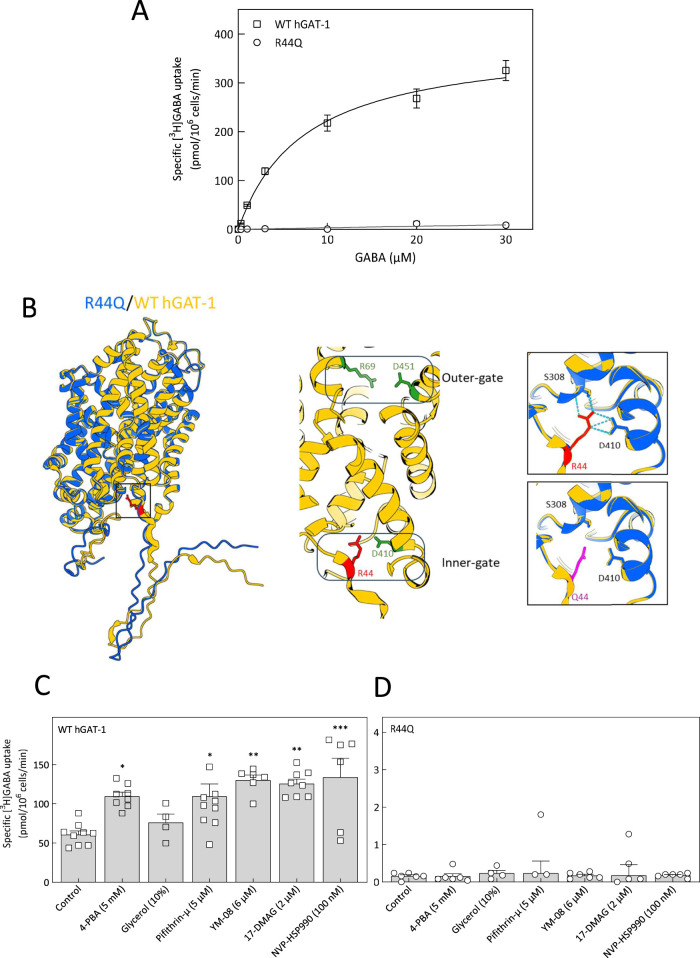
Structural and functional analysis of the R44Q variant and small molecule screening *in vitro*. **(A)** Michaelis-Menten [^3^H]GABA uptake kinetics of WT hGAT-1 and R44Q mutant was carried out in transiently transfected HEK293 cells. Specific [^3^H]GABA uptake was measured for exactly 3 min, as explained in the “Methods” section. Non-specific uptake was defined in the presence of 10 µM tiagabine. The kinetic parameters, K_m_ and V_max_ values, were determined for WT hGAT-1 only. **(B)** Protein structure predictions for the R44Q variant were generated using ColabFold v1.5.5 (Mirdita et al., 2022). The left panel shows the computed ribbon structure of R44Q (blue) superimposed on the wild-type GAT-1 structure (yellow; AlphaFold ID: AF-P30531-F1-v4). Residue 44 is indicated as bright red sticks and highlighted in a black box, which is magnified in the right panels. The middle panel highlights the outer gate (residues R69 and D451, shown as green sticks) and the inner gate (residues R44, shown in red, and D410, shown in green) of GAT-1. The right panel presents a magnified view of R44 (top, red) and Q44 (bottom, magenta). Blue dotted lines indicate hydrogen bonds. **(C,D)** HEK293 cells were transiently transfected with plasmids driving the expression of YFP-tagged WT hGAT-1 and R44Q mutant. Post 24 h, the cells were seeded onto PDL-coated 48-well plates and treated with the indicated drugs (4-PBA (5 mM), glycerol (10%), pifithrin-µ (5 µM), YM-08 (6 µM), 17-DMAG (2 µM) and NVP-HSP990 (100 nM)) for following 24 h. Specific [^3^H]GABA uptake (single concentration) was measured for exactly 3 min, as described under “Methods”. The data were obtained from at least three independent experiments, performed in triplicate (error bars represent S.E.M). The data sets were statistically compared by one-way ANOVA, followed by Tukey´s *post hoc* tests (*, p < 0.05, **, p < 0.01, ***, p < 0.001).

Arginine 44 (R44) of human GAT-1 is evolutionarily conserved across the SLC6 transporter family, including in the bacterial leucine transporter (LeuT) (Table 1). Mutations at the equivalent arginine residue in other SLC6 members have been implicated in various human diseases (Table 2). To investigate the functional consequences of epilepsy-associated mutations at this conserved position, we assessed the activity of wild-type (WT) hGAT-1 and the R44 variants (R44Q, R44P, and R44W). We performed single-point radioligand uptake assays in HEK293 cells transiently transfected with WT or mutant hGAT-1. As shown in Figure 1A, none of the three epilepsy-associated mutants exhibited appreciable GABA uptake, indicating severe functional impairments. To determine whether the loss of function was due to trafficking defects, we analyzed the glycosylation status of the mutant proteins using endoglycosidase H (Endo H). GAT-1 is glycosylated at multiple sites during biosynthesis and maturation. Core-glycosylated forms retained in the endoplasmic reticulum (ER) are sensitive to Endo H, whereas maturely glycosylated proteins that reach the plasma membrane are Endo H-resistant due to modifications acquired in the Golgi. In WT hGAT-1, two bands were detected (Figure 1B, left panel): a lower band that was Endo H-sensitive, representing ER-localized core-glycosylated forms, and an upper band that was Endo H-resistant, corresponding to mature transporters at the plasma membrane. We quantified the mature band intensities of the YFP-tagged transporters wherein all three functionally-deficient mutants (R44Q, R44P, R44W) displayed substantial levels of the Endo H-resistant band, suggesting that these proteins reached the cell surface in the same manner as the WT hGAT-1 (Figure 1B, right panel). To confirm the subcellular localization of the mutant transporters, we conducted confocal laser scanning microscopy in HEK293 cells expressing YFP-tagged GAT-1 constructs. Co-staining with CFP-tagged calnexin (an ER marker) and trypan blue (a plasma membrane marker) revealed that the YFP-tagged R44 mutants localized predominantly to the plasma membrane (Figure 1C, second to fourth rows), analogous to WT hGAT-1 (top panel). These localization patterns corroborated the glycosylation profiles observed. Collectively, these data indicate that the R44 mutants are correctly folded, trafficked through the secretory pathway and inserted into the plasma membrane - yet they are non-functional at their site of action. This observation is consistent with our previous work on other SLC6 transporters, which suggest that stabilization of the inward-facing conformation during ER quality control is critical for proper membrane targeting, though it does not guarantee functional competency at the cell surface (Kasture et al., 2017). These findings suggest that the functional abolition in the R44 variants may arise from conformational alterations that disrupt important steps in the GABA transport cycle, such as substrate binding or translocation. To assess the precise impact of the R44Q mutation on GABA transport kinetics, we performed a radioligand uptake assay across a range of GABA concentrations to generate Michaelis–Menten kinetics curves (Figure 2A). While WT hGAT-1 displayed saturable uptake characteristic of fully functional transporter activity, the R44Q mutant exhibited no detectable GABA uptake, confirming a complete abolition of GABA transport (the K_m_ and V_max_ values determined for the WT transporter were: 7.5 ± 1.3 µM and 387.7 ± 23.4 pmol/10^6^ cells/min, respectively).

**TABLE 2:** Disease-associated variants at R44 in hGAT-1 and at corresponding residues in other SLC6 transporters.

Gene	Name	Variant	Disease links (predictions)	Missense score	Uniprot ID
*slc6a1*	hGAT1	R44Q	Myoclonic-atonic epilepsy	0.99	rs794726859
		R44P	Myoclonic-atonic epilepsy	1	rs794726859
		R44W	Myoclonic-atonic epilepsy	1	rs1553687863
*slc6a3*	hDAT	R60W	Classic dopamine transporter deficiency syndrome, likely pathogenic	0.91	rs1579729357
*slc6a5*	hGlyT2	R191G	Hyperekplexia 3, pathogenic	1	rs376783257
*slc6a19*	hB^0^AT1	R32Q	Neutral 1 amino acid transport defect, variant of uncertain significance	0.41	rs190631924

Pathogenicity predictions were generated using AlphaMissense (Cheng et al., 2023), with missense scores indicating the likelihood of each variant being deleterious.

To gain structural insights into the effects of the R44Q mutation, we employed AlphaFold two to predict the conformational alterations introduced by this substitution (Mirdita et al., 2022). Figure 2B, shows the predicted R44Q (blue) structure superimposed on WT hGAT-1 (yellow). Protein structure prediction for R44Q did not divulge any major structural alterations. The middle panel displays a partial ribbon diagram of WT hGAT-1, highlighting the outer (R69 and D451) and inner gate residues (R44 and D410). R44 is positioned adjacent to TMD1 and forms hydrogen bonds with residues S308 and D410 (Figure 2B, magnified boxes). These interactions are absent in the glutamine 44 (R44Q) mutant. However, the N-terminus predominantly appears as an unstructured ribbon in the AlphaFold two model, suggesting this region is intrinsically disordered and associated with lower prediction confidence; similar observations have been made for the serotonin transporter (SERT) using an approach that combines molecular modeling, circular dichroism spectroscopy and Förster Resonance Energy Transfer (FRET) microscopy (Fenollar-Ferrer et al., 2014). We also employed three support vector machine (SVM)-based algorithms to calculate ΔΔG values, which are indicative of changes in structural stability (Capriotti et al., 2005; Chen et al., 2004; Savojardo et al., 2016). As shown in Table 3, all three SVM algorithms predict that the mutation at position 44 leads to a reduction in protein stability.

**TABLE 3:** *In silico* prediction of protein stability changes for R44 variants using multiple computational tools.

Mutation	MUpro	I-Mutant v2.0	INPS-MD
	*ΔΔG value*	*Stability*	*ΔΔG value*
R44Q	−1.2967405	Decrease	−1.29 (destabilizing)
R44S	−0.8725703	Decrease	−1.19 (destabilizing)
R44W	−1.3183621	Decrease	−0.37 (neutral)
R44P	−1.6349691	Decrease	−1.33 (destabilizing)

### 3.2 Rescue of R44Q using strategies targeting multiple checkpoints in membrane trafficking

**FIGURE 3:**
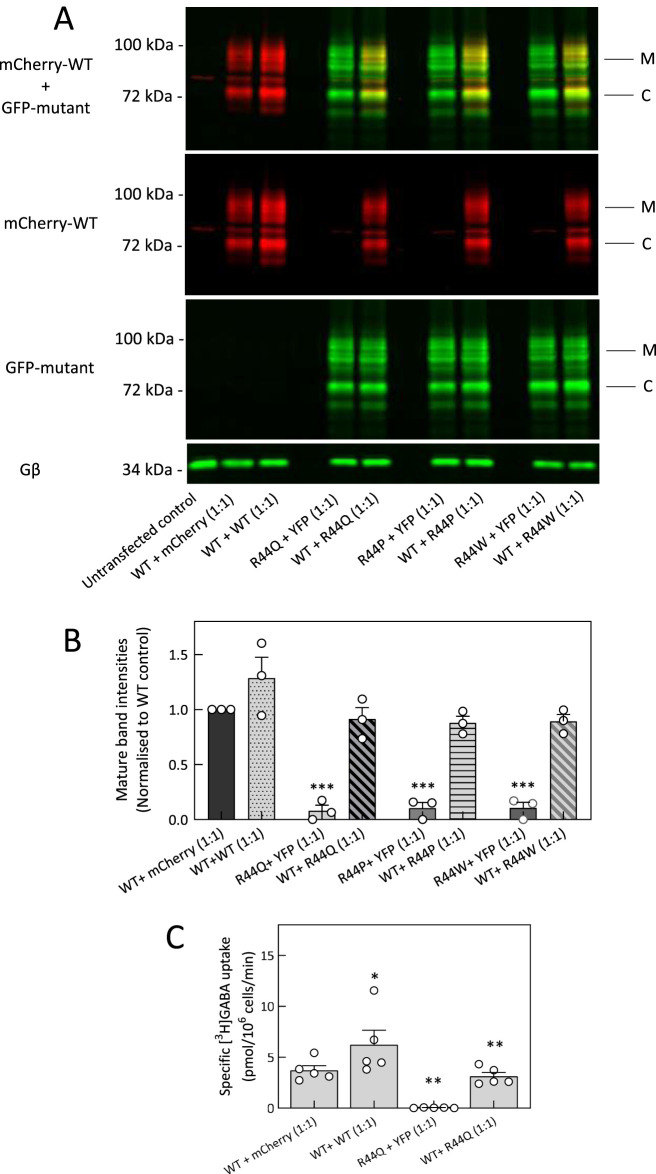
R44 mutants do not alter the trafficking and activity of WT hGAT-1. **(A)** mCherry-tagged WT hGAT-1 and YFP-tagged R44 mutants were co-transfected in HEK293 cells at a 1:1 plasmid ratio, to differentiate between the expression of WT (red channel) and mutants (green channel). Untransfected HEK293 cells served as negative controls. Cell lysates were prepared 48 h post transfection, resolved by gel electrophoresis and probed with GFP and mCherry antibodies as described under “Methods”. The upper panel is a merged image of mCherry-WT hGAT-1 and YFP-tagged R44 mutants. M and C signify the mature-glycosylated and core-glycosylated protein species, respectively. The middle and bottom panels are individual blots for mCherry-WT (red) and YFP-tagged mutants (green), respectively, at identical fluorescence settings on Odyssey CLx. The mCherry-WT hGAT-1, YFP-R44Q, YFP-R44P and YFP-R44W were immunoprecipitated using respective primary antibodies probed for mCherry and GFP. Subsequently, fluorescence was detected by secondary antibodies against mouse mCherry and rabbit GFP. R44 mutants showed no effect on WT mature glycosylated bands (red bands, comparable to WT control). **(B)** Quantification of mature band intensities of mCherry-WT, upon co-transfection with R44Q, R44P and R44W mutants at the 1:1 plasmid ratio. The mature bands of mCherry-WT hGAT-1 were normalized to Gβ and plotted relative to the control (WT + mCherry) condition. The data were obtained from three independent experiments (error bars = SEM). The normalized values were statistically compared by one-way ANOVA, followed by Tukey´s *post hoc* t-tests (***, p < 0.001). **(C)** Radioligand [^3^H]GABA uptake assay was performed in HEK293 cells expressing mCherry-tagged WT hGAT-1 and YFP-tagged R44Q mutant together at 1:1 plasmid ratio (by weight) to evaluate the transport activity of WT hGAT-1 upon co-expression. YFP and mCherry empty vectors were used to ensure equal DNA amounts in all conditions. Specific GABA uptake was measured over a 3-min period, with non-specific uptake determined in the presence of 10 µM tiagabine. The WT transport activity did not change upon co-expression with R44Q. Data represent means ± SEM, from at least three independent experiments performed in triplicate. Statistical significance was assessed by one-way ANOVA followed by Tukey´s *post hoc* tests (*, p < 0.05, **, p < 0.01).

Protein folding and surface expression can be promoted by chemical and pharmacological chaperones. This strategy not only enhances the expression of WT SLC6 transporters, but also facilitates the functional rescue of misfolded/trafficking-deficient mutant proteins (Sucic et al., 2016; Kasture et al., 2017). The chemical chaperone 4-PBA - which is known to prevent aggregation of misfolded proteins, downregulate HSP70 activity (Rubenstein and Lyons, 2001) and attenuate ER stress–efficiently corrected folding-impaired disease variants of the creatine transporter one and the organic cation transporter 3 (El-Kasaby et al., 2019; Angenoorth et al., 2022). In addition, our recent studies bared 4-PBA as a potential therapeutic compound in rescuing hGAT-1 epilepsy variants *in vitro* and *in vivo* (Kasture et al., 2023; Shah et al., 2025). In the present study, 4-PBA did not improve the GABA uptake activity of the loss-of-function R44Q mutant (Figure 2D, *second bar*). Treatment with 10% glycerol - another chemical chaperone, which was shown to induce the expression of the ΔF508-CFTR mutant (Sato et al., 1996) and a recently investigated partially-misfolded G443D-hGAT-1 mutant (Shah et al., 2025) – was also ineffective on R44Q (Figure 2D, *third bar*). We employed an additional mechanistic strategy to assist R44Q, by blocking the heat shock protein (HSP) relay in order to relax the ER quality control machinery, which previously proved promising on DAT and SERT (El-Kasaby et al., 2014; Mazhar Asjad et al., 2017). As expected, this approach also failed to show any promising effect on the surface-bound, but inactive, R44Q - regardless of which HSP isoform was inhibited (i.e., pifithrin-µ and YM-08 for HSP70 or 17-DMAG and NVP-HSP990 for HSP90; Figure 2D). Treatment led to a marked increase in WT transporter activity (Figure 2C), suggesting that improved surface expression directly contributes to the observed improvement in GABA uptake. Mutations in SLC6 transporters have been reported to follow both autosomal dominant and autosomal recessive modes of inheritance (Chiba et al., 2014). We tested whether R44 mutants exert dominant-negative effects on WT hGAT-1, using a co-expression protocol with fluorescently tagged constructs (mCherry-WT and YFP-mutants) in HEK293 cells. Co-transfection of WT with R44Q, R44P, or R44W (1:1 plasmid ratio) showed no reduction in surface expression or trafficking of WT compared to control (Figures 3A,B). Radiolabeled GABA uptake assays further demonstrated that R44Q did not impair WT transport activity when co-expressed (Figure 3C). Thus, R44 mutants do not act in a dominant-negative manner on WT hGAT-1. Consistent with our previous findings on other *SLC6A1* variants, these results suggest that properly trafficked mutants exhibit reduced stability or function due to underlying conformational defects, whereas heterozygous loss-of-function effects likely result from haploinsufficiency.

**FIGURE 4:**
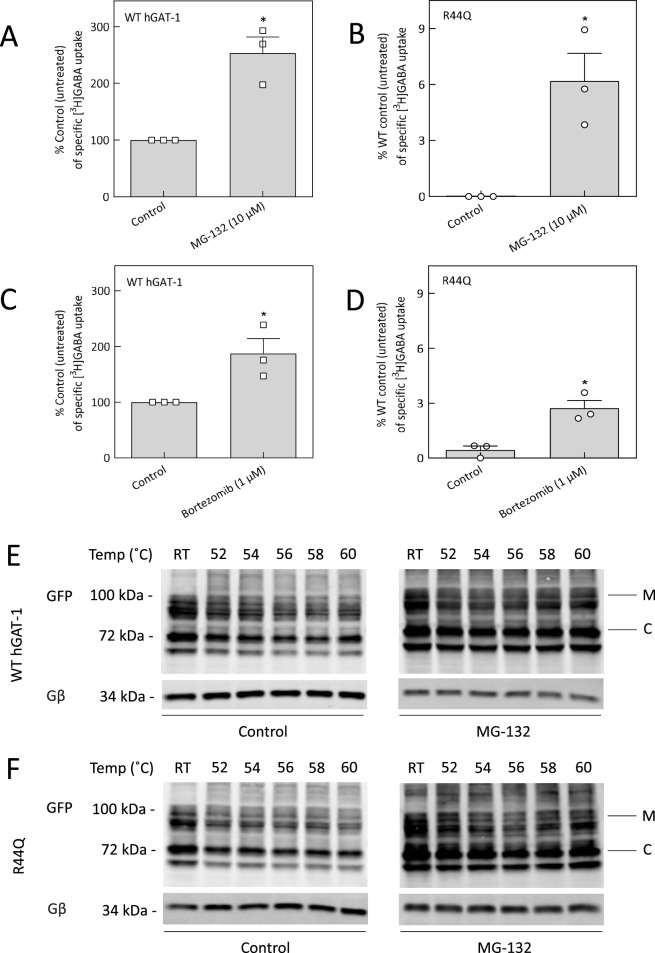
Proteasome inhibition rescues the uptake activity and thermal stability of R44Q. **(A–D)** HEK293 cells transiently expressing YFP-tagged WT hGAT-1 or R44Q mutant were seeded onto PDL-coated 48-well plates 24 h post-transfection and treated with proteasome inhibitors MG-132 (10 µM) or bortezomib (1 µM) for an additional 24 h. Specific [^3^H]GABA uptake was measured for exactly 3 min, as described in Methods. Data represent mean ± SEM from at least three independent experiments performed in triplicate. Statistical significance was assessed by paired t tests (*p < 0.05). **(E, F)** Representative immunoblots showing the thermal stability profiles of WT hGAT-1 and R44Q mutant at increasing temperatures, before and after MG-132 treatment (10 µM).

We hypothesized that the non-functional R44Q mutant, despite its localization to the plasma membrane, may undergo intracellular degradation via an unidentified pathway. To explore this possibility, we targeted the proteasomal degradation system—a key mechanism for maintaining cellular proteostasis through the clearance of polyubiquitin-tagged proteins (Skalniak et al., 2013). HEK293 cells expressing the R44Q mutant were treated *in vitro* with the proteasome inhibitors MG-132 (10 µM) and bortezomib (1 µM), and their effects were assessed (Figures 4A–D). Interestingly, MG-132 treatment led to an approximate 8% increase in GABA uptake activity of the R44Q mutant relative to the WT hGAT-1 control (Figure 3B), while bortezomib resulted in only a marginal functional rescue (Figure 4D). Given the superior efficacy of MG-132, we further investigated its mechanism of action by evaluating the thermal stability of the R44Q mutant before and after MG-132 treatment (Figures 4E,F). Upon treatment, the mature glycosylated bands of R44Q at the plasma membrane showed enhanced thermal stability across a temperature range of 52 °C–60 °C (Figure 4F, right panel), compared to the untreated condition (Figure 4F, left panel).

**FIGURE 5:**
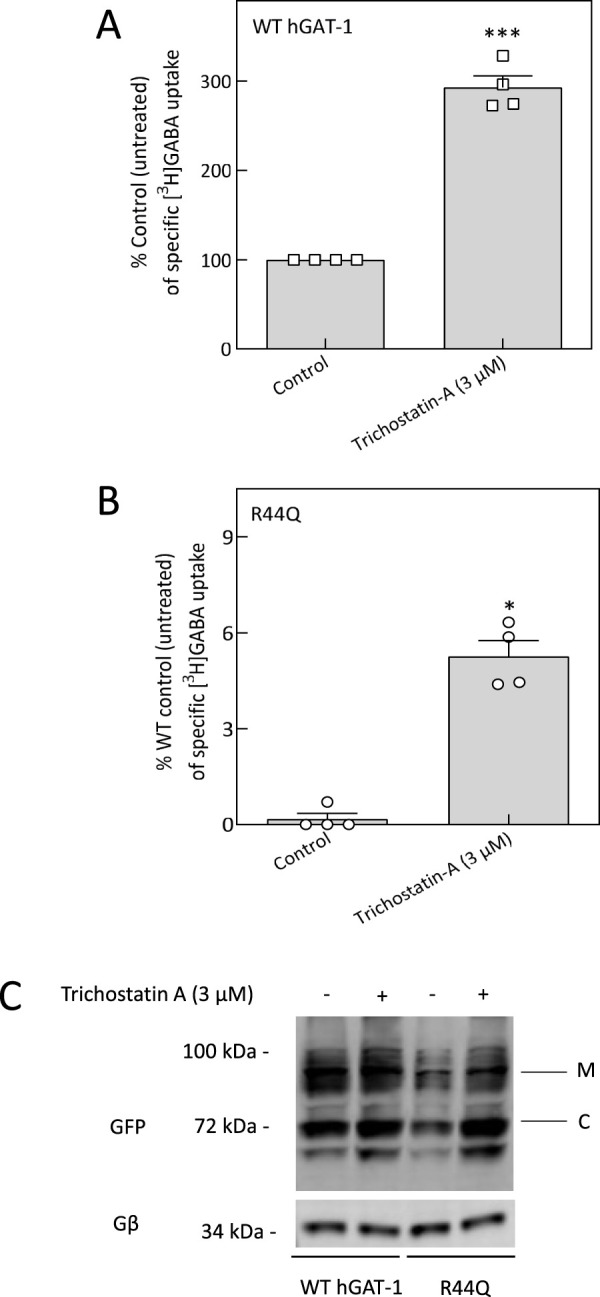
Functional rescue of R44Q by trichostatin A treatment. **(A,B)** HEK293 cells transiently expressing YFP-tagged WT hGAT-1 or R44Q mutant were seeded onto PDL-coated 48-well plates 24 h post-transfection and treated with trichostatin A (3 µM) for 24 h. Specific [^3^H]GABA uptake was measured over 3 min, as detailed in the Methods. Data represent mean ± SEM from at least three independent experiments performed in triplicate. Statistical analysis was performed using paired t tests (*p < 0.05, ***p < 0.001). **(C)** Representative immunoblots of WT and R44Q hGAT-1 before and after trichostatin A treatment. Bands corresponding to mature glycosylated (“M”) and core-glycosylated (“C”) forms are indicated in the figure panel.

Microtubules have been shown to play a role in both the trafficking and regulation of SLC6 transporters (Wersinger and Sidhu, 2005; 2009; Jeannotte and Sidhu, 2007; Jeannotte et al., 2009; Fournet et al., 2010). Accordingly, we further examined the role of microtubule dynamics in modulating the trafficking and function of the R44Q hGAT-1 mutant. Microtubules are characterized by considerable chemical and functional heterogeneity, with dynamic regulation of their composition, post-translational modifications, and interactions with associated proteins (Sferra et al., 2020). Given the significance of α-tubulin acetylation as a hallmark of stabilized microtubules, we focused on manipulating this modification to assess its impact on mutant transporter behavior (Zilberman et al., 2009). To this end, we used trichostatin A (TSA), a potent inhibitor of histone deacetylase 6 (HDAC6), which is responsible for α-tubulin deacetylation (Zilberman et al., 2009). HEK293 cells expressing the R44Q mutant were treated with 3 μM TSA, and the effects were evaluated on transporter function and localization (Figure 5). TSA treatment resulted in an apparent, though statistically insignificant, increase in radiolabeled GABA uptake (Figure 5B), accompanied by an approximately 1.4-fold rise in the surface expression of the R44Q mutant protein (Figure 5C; Supplementary Figure S1).

### 3.3 Seizure susceptibility and differential expression of the R44Q variant in fruit flies

**FIGURE 6:**
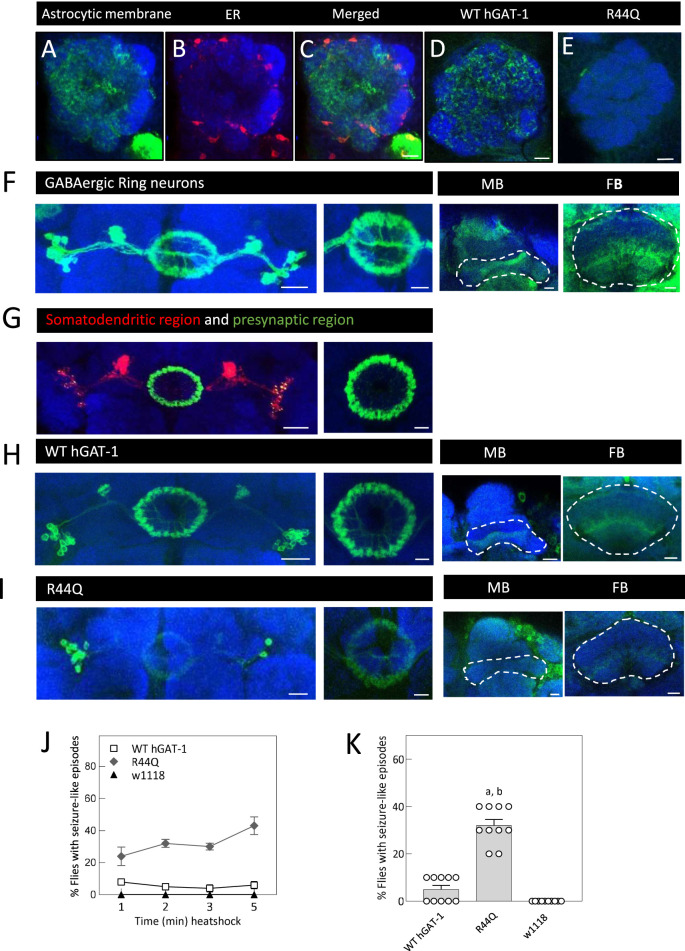
Seizure-like phenotypes and cell-type-specific expression of the R44Q variant in *Drosophila*. **(A–I)**, confocal images were acquired of adult fly brains. Magnified view of a section of antennal lobe (AL) showing astrocytic membranes **(A,C)** and ER **(B,C)** (*; UAS-mCD8 GFP/ALRM Gal4; UAS-RFP.KDEL/+*), expression of WT-hGAT-1 **(D)** (*; ALRM Gal4; UAS-YFP-hGAT-1 WT*) and R44Q **(E)** (*; ALRM Gal4; UAS-YFP-hGAT-1-R44Q*). AL neuropile is visualized with Neural cadherin (NCAD in blue). **(A–E)** Scale bar = 10 µm. **(F)** GABAergic ring neurons were visualized using CD8 membrane marker fused to GFP *(; R20D01-p65.AD/UAS mCD8-GFP; R72B07-GAL4.DBD/+*). Right hand panel shows a magnified view of the ellipsoid body, Mushroom body (MB) lobes and Fan-shaped body (FB) lobes. MB and FB lobes were labeled using GAD-1 Gal4 (*; UAS-mCD8 GFP/Gad-1 Gal4;* ) **(G)** Visualization of somatodendritic and presynaptic compartments of GABAergic ring neurons (*; UAS-DenMark, UAS-Syt-GFP; R20D01-p65.AD; R72B07-GAL4.DBD/+*). **(H,I)** Expression of WT-hGAT1 and R44Q in ring neurons and GAD-1 neurons, respectively (*R20D01-p65.AD/+; UAS-YFP hGAT-1-WT/R72B07-GAL4.DBD and R20D01-p65.AD/+; UAS-YFP hGAT-1-R44Q/R72B07-GAL4.DBD*). MB and FB lobes were labeled using GAD-1 Gal4 (*;Gad-1 Gal4/+; UAS-YFP hGAT-1-WT/+;* and (*;Gad-1 Gal4/+; UAS-YFP hGAT-1-R44Q/+*) Scale bars **(F–I)**: left panel = 20 μm, right panel = 10 µm. **(J,K)** Seizure susceptibility in said genotypes was studied. Briefly, heat induced seizures were studied in 10 three-to-five-day old male R44Q (*+/y; ALRM Gal4; UAS-YFP-hGAT-1-R44Q*) WT (*+/y; ALRM Gal4; UAS-YFP-hGAT-1 WT*) and *w* ^*1118*^ at 1-, 2-, 3- and 5-min intervals. **(K)** Percent flies showing seizure-like activity, following 2 min heat induction. Data shown in mean ± SEM. The statistical comparison was done by analysis of variance followed by Dunn’s *post hoc* tests (p value for WT compared to R44Q **(A)** = 0.0019, p value for w1118 compared to R44Q **(B)** = <0.0001). Endogenous dGAT was not visualized in the confocal images.

**FIGURE 7:**
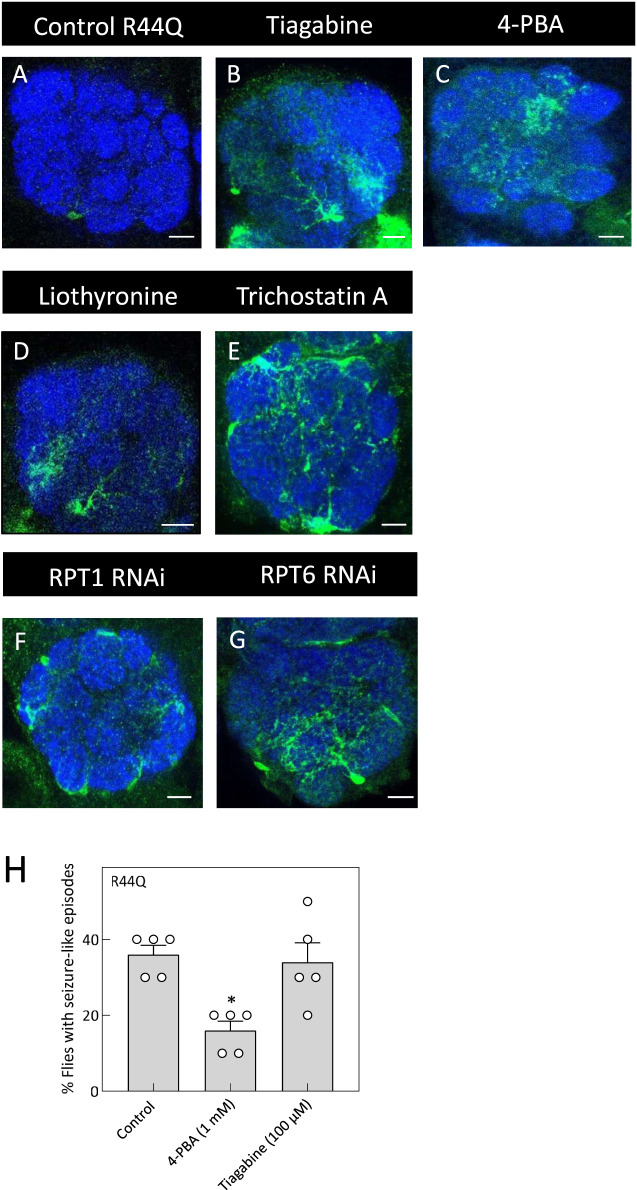
*In vivo* rescue of the R44Q variant in flies. **(A)** Expression of R44Q (*+/y; ALRM Gal4; UAS-YFP-hGAT-1-R44Q*) in the AL neuropile in either untreated flies or 100 μM tiagabine **(B)**, 1 mM 4-PBA **(C)**, 100 μM liothyronine **(D)** or 1 mM trichostatin A **(E)** treated flies. **(F,G)** Expression of R44Q in astrocytes, co-expressed with RPT1 RNAi **(F)** (*+/y; ALRM Gal4; UAS-YFP-hGAT-1-R44Q/UAS-RPT RNAi*) and co-expressed with RPT6 RNAi **(G)** (*+/y; ALRM Gal4; UAS-YFP-hGAT-1-R44Q/UAS-RPT RNAi*). Scale bar = 10 µm. **(H)** Three-to-five day old male R44Q flies (*+/y; ALRM Gal4; UAS-YFP-hGAT-1-R44Q*) were treated with 1 mM 4-PBA and 100 μM tiagabine and their seizure susceptibility was studied. Data shown in mean ± SEM. The statistical comparison was done by analysis of variance followed by Dunn’s *post hoc* tests (p value for untreated compared to 4-PBA (*) = 0.0172). Endogenous dGAT was not visualized in the confocal images.

We next characterized the R44Q mutant in *D. melanogaster*. Our recent studies, relying on fruit flies, have focused on protein folding, trafficking and activity of other SLC6 transporters - such as DAT and SERT (Kasture et al., 2016; 2019; 2023; Bhat et al., 2017; 2021; 2023; Mazhar Asjad et al., 2017; El-Kasaby et al., 2024; Shah et al., 2025). In *Drosophila*, GAT expression is restricted to astrocytes (Muthukumar et al., 2014). We used the phiC31 integrase system to generate transgenic flies expressing the YFP-tagged human hGAT-1-R44Q variant (Bischof et al., 2007); with the insertion site kept consistent with that of the WT hGAT-1, to control for positional effects (Kasture et al., 2023). Previously, we analyzed hGAT expression in the antennal lobe (AL) neuropil, where astrocytes are located peripherally and extend fine processes toward the interior (Kasture et al., 2023; Shah et al., 2025). We visualized reporter expression using the astrocyte-specific ALRM GAL4 driver (Doherty et al., 2009). Figure 6A shows astrocytic membranes labeled with GFP fused to murine CD8 (mCD8), and the endoplasmic reticulum (ER) marked with RFP fused to a KDEL sequence in a single AL optical plane (Figures 6B,C). WT hGAT-1 exhibited broad expression closely matching the membrane marker pattern (Figure 6D), while R44Q expression was markedly reduced in the AL (Figures 6E, 7A).

We also examined neuronal expression of R44Q in GABAergic ellipsoid body (EB) neurons and GAD-1–positive neurons (Certel et al., 2022). EB neurons are bilaterally symmetric, with lateral cell bodies projecting to the central ring neuropil (Figure 6F). The broad GABAergic neuron driver GAD-1 GAL4 labels innervations into presynaptic mushroom body (MB) and fan-shaped body (FB) neuropils (Figure 6F). Somatodendritic compartments of EB neurons were visualized using mCherry fused to the somatodendritic marker ICAM5, while GFP fused to synaptotagmin marked presynaptic terminals in the central ring (Figure 6G) (Nicolaï et al., 2010). WT hGAT-1 was expressed in both somatodendritic and presynaptic compartments of GABAergic EB neurons (Figure 6H), with additional expression in MB and FB neuropils. By contrast, the R44Q mutant showed clear expression in both somatodendritic and presynaptic regions of EB neurons (Figure 6I), but only low expression in the FB, and little to no detectable expression in the MB neuropil (Figure 6I).

Flies expressing disease-associated hGAT-1 variants exhibit heat-induced seizure-like behavior (Kasture et al., 2023; Shah et al., 2025). This phenotype is characterized by increased locomotion, uncoordinated movements, leg twitching, and wing flapping, followed by a loss of posture and temporary paralysis. Flies typically recover and regain normal posture after a brief period. To evaluate the *in vivo* functional consequences of the R44Q mutation, 3- to 5-day-old male flies expressing YFP-tagged hGAT-1 R44Q were subjected to a heat-induced seizure assay. Flies were transferred to plastic vials and immersed in a water bath maintained at 40 °C for 5 min. Flies expressing YFP-tagged WT hGAT-1 and w1118 served as controls. As shown in Figure 6J, R44Q flies displayed clear seizure-like behavior. Seizures were first observed as early as 20 s into the assay, with approximately 30% of flies showing susceptibility by 2 min (Figure 6K), increasing to ∼43% by the end of the 5-min exposure. In contrast, only ∼10% of WT flies showed mild seizure-like activity, while w1118 control flies exhibited no seizure susceptibility under parallel conditions.

To test whether chemical and pharmacological chaperones could restore astrocytic expression of R44Q (Figure 7A), flies were given food supplemented with tiagabine (Figure 7B), 4-PBA (Figure 7C), or liothyronine (Figure 7D). As shown in Figures 7A–D, all three compounds considerably increased R44Q expression in the antennal lobe. Additionally, treatment with trichostatin A (TSA) led to increased expression of the R44Q mutant in the antennal lobe (AL) neuropil. To further investigate the role of proteasomal degradation in regulating mutant transporter levels, we employed RNA interference (RNAi) to knock down the proteasomal subunits RPT1 and RPT6. Silencing of either subunit resulted in a marked restoration of R44Q expression in astrocytes. As shown in Figures 7F,G, RNAi-mediated knockdown of RPT1 or RPT6 significantly enhanced astrocytic expression of the R44Q variant, supporting the role of proteasomal pathways in modulating its stability and accumulation. We also evaluated whether pharmacological interventions can mitigate seizure-like activity observed in R44Q flies. Flies with astrocyte-specific R44Q expression were treated for 48 h with 1 mM 4-PBA (a chemical chaperone known to promote protein folding) or with 100 µM tiagabine (a clinically used GAT-1 inhibitor). As shown in Figures 7H, 4-PBA treatment significantly reduced seizure susceptibility in R44Q flies, whereas tiagabine had no evident effect. These results suggest that improving protein expression can be an effective strategy for alleviating certain mutant-associated seizure phenotypes.

## 4 Discussion

The *SLC6A1* gene demonstrates particular vulnerability to missense mutations, which can disrupt transporter function through diverse mechanisms, including impaired folding, trafficking, or substrate translocation (Silva et al., 2024). The majority of these variants accumulate intracellularly in both HEK293 cells and *D. melanogaster*, although some respond to pharmacochaperone-mediated rescue (Kasture et al., 2023; Shah et al., 2024; 2025). Here, we observed that - although 4-PBA did not restore GABA transport in R44Q-expressing HEK293 cells - it not only increased the expression of R44Q in *Drosophila* astrocytes, but also ameliorated the seizure-like behavior. The precise reasons for the discrepancy between the effects of 4-PBA observed *in vitro* and *in vivo* remain unclear; however, it is plausible that certain factors inherent to the *in vivo* environment contribute to the observed differences. 4-PBA has been reported to modulate the expression of various molecular chaperones (including calreticulin, Hsc70, and DnaJ-like one and 2) as well as metabolic enzymes in *Drosophila* (Kang et al., 2002). A clinical trial is currently assessing glycerol phenylbutyrate (Ravicti) in patients with GAT-1- and STXBP1-related developmental and epileptic encephalopathies (Grinspan et al., 2024). Our *in vivo* data indicate that 4-PBA may hold therapeutic potential for mitigating R44Q-mediated pathologies. ER-targeted 4-PBA derivatives have shown greater efficacy than 4-PBA alone in a *Drosophila* model of ALS, likely due to their direct engagement with the ER (Azoulay-Ginsburg et al., 2021). These targeted approaches may help expand the therapeutic utility of 4-PBA for SLC6A1-and related disorders.

Unlike the G443D variant, which was rescued by the chemical chaperone glycerol (Shah et al., 2025), the surface-bound R44Q mutant showed no response to protein-stabilizing effects of this small molecule. SLC transporters are subject to efficient and rapid degradation via the proteasomal pathway (Bensimon et al., 2020). The proteasome degrades proteins, including those destined for the cell surface, which controls the number of these proteins present at any given time. The proteasome can be localized to the cell surface or associated with surface proteins in processes like endocytosis, which is essential for signaling and other functions. We explored proteasome inhibition as an alternative rescue strategy. In *Drosophila* astrocytes, primarily expressing the GAT, knockdown of proteasomal subunits Rpt1 and Rpt6 augmented R44Q expression. Treatment with MG-132 increased GABA uptake and surface expression of R44Q, while bortezomib had a minimal effect. Proteasome-based stabilization has been observed in other models, including the restoration of dystrophin expression in mdx muscle (Bonuccelli et al., 2003) and increased MCPIP1 expression in human cells (Skalniak et al., 2013). These results indicate that the proteasome may represent a relevant target for further study in SLC6A1 disorders. In addition, microtubule dynamics contribute to the regulation of GAT-1 trafficking. Post-translational modifications, such as acetylation, promote motor protein binding and cargo transport (Zilberman et al., 2009). TSA treatment increased both the expression and GABA uptake activity of the R44Q mutant, a finding that was further validated in astrocytes of R44Q transgenic flies. Stabilized, acetylated microtubules may therefore enhance the trafficking and/or retention of the mutant transporter at the plasma membrane. Mechanistically, the rescue could arise from one or more factors: enhanced kinesin-1 binding to acetylated microtubules, more effective trafficking, or increased stability of surface-localized protein mediated by interactions with SNARE complexes. Surprisingly, distinct microtubule modifications had differential outcomes. Blocking detyrosination with parthenolide failed to rescue the R44Q mutant (data not shown), underscoring the importance of specific post-translational modifications in controlling transporter trafficking and function. TSA was shown to significantly increase DAT and SERT transcript levels in SK-NF-I human neuroblasts (Bence et al., 2012); but, its potential effects on hGAT-1 remain to be established.

In both cellular and *Drosophila* models, the well-characterized disease-relevant hGAT-1 variants, A288V, G443D and G443V, demonstrated consistent trends with neuronal and astrocytic expression patterns closely aligning with the *in vitro* effects (Kasture et al., 2023; Shah et al., 2025). As an example, the trafficking-deficient A288V mutant was retained in the ER compartment in both HEK293 cells and in GABAergic neurons and astrocytes of *Drosophila* (Kasture et al., 2023). These observations were made against the background of unlabeled astrocytic dGAT. It is also important to note that HEK293 cells transiently express the GAT-1, whereas in *Drosophila*, the protein is expressed continuously throughout both larval and adult stages (Huang et al., 2015). Humans possess four genes encoding distinct GABA transporters responsible for GABA reuptake, whereas *Drosophila* express only a single gene encoding GAT (Muthukumar et al., 2014). As endogenous dGAT is limited to astrocytes, fruit flies provide a unique situation where R44Q expression can be visualized without the influence of dGAT (i.e., in neurons) and in presence of dGAT (i.e., in astrocytes). Patients suffering from SLC6A1 pathologies display haploinsufficiency and harbor one copy with the mutation. Transgenic flies expressing astrocytic R44Q used in these studies also expressed endogenous astrocytic GAT, which - at least partially - mimics the heterozygous clinical setting. Drawing on earlier hGAT-1 mutant expression data, one might have predicted that the R44Q variant would localize to the cell surface in both neurons and glia. However, R44Q displayed diminished protein levels within the presynaptic compartments of GABAergic neurons. R44Q expression was substantially reduced in astrocytes, with the remaining signal predominantly restricted to glial cell bodies. The G443D mutant, previously reported to exhibit ample surface expression in transfected HEK293 cells, had astrocytic expression comparable to that of WT hGAT-1 (Shah et al., 2025). Knockdown of proteasomal genes, though, led to increased R44Q expression in astrocytes, suggesting rapid proteasomal degradation. This effect was not observed for A288V, highlighting variant-specific differences in degradation or quality control mechanisms among individual SLC6A1 mutant proteins (Kasture et al., 2023).

Despite sequence variation, the cytoplasmic N-terminus of SLC6 transporters retains conserved structural features that are essential for transporter function (Baliova and Jursky, 2024). The internal gate of GAT-1 regulates GABA flux (Dayan-Alon and Kanner, 2019), and mutations in this domain tend to clench the transporter in an inward-facing state (Ben-Yona and Kanner, 2013; Kniazeff et al., 2008). Conformational changes arise from movements between the bundle domain (TMDs 1, 2, 6, 7) and scaffold domain (TMDs 3-5, 8-10), coordinating the opening and closing of extracellular and intracellular gates (Yamashita et al., 2005; Krishnamurthy and Gouaux, 2012; Zhu et al., 2023). Vital salt-bridge interactions, including those between R60, D436, and Y335 in related SLC6 transporters, such as DAT, play a crucial role in stabilizing these conformations (Kniazeff et al., 2008). In hGAT-1, the positively charged R44 forms a similar salt bridge with D410 in TMD8, crucial for GABA transport (Ben-Yona and Kanner, 2013). The conserved R^44^XXW^47^ motif is, in fact, essential for transporter stability and function (Bennett et al., 2000) and R44 mutations are likely to perturb the aforementioned interactions, resulting in protein destabilization and impaired GABA uptake, stressing the significance of the N-terminal region. In a comparable mechanism, phosphorylation of N-terminal residues in DAT is required for amphetamine-stimulated dopamine efflux (Khoshbouei et al., 2004). Moreover, in other monoamine transporters, such as SERT, the N-terminus facilitates amphetamine-induced substrate efflux by stabilizing the transporter in a conformation that supports this function (Sucic et al., 2010; Kern et al., 2017). This also involves interactions with phosphatidylinositol-4,5-bisphosphate (PIP2) (Buchmayer et al., 2013; Hamilton et al., 2014; Sitte and Freissmuth, 2015; Bermingham and Blakely, 2016; Luethi et al., 2022), syntaxin-1A (Deken et al., 2000), protein kinase C (PKC) (Beckman et al., 1998), and calpains (Baliova et al., 2009; González, 2019). Hence, numerous factors collectively contribute to and influence SLC6 transporter function and membrane dynamics.

## 5 Summary and concluding remarks

*SLC6A1* is highly susceptible to missense mutations that induce conformational perturbations, compromising the localization and functional integrity of hGAT-1. In this study, we delineate the molecular and functional consequences of mutations at the evolutionarily conserved arginine residue R44 of hGAT-1, which are clinically-linked to epilepsy phenotypes. Positioned within the critical RXXW motif, R44 constitutes a key component of the inner gate of the substrate translocation pathway via its interaction with residue D410. Our findings demonstrate that epilepsy-associated variants R44Q, R44P and R44W retain plasma membrane trafficking but are functionally impaired. Computational stability predictions, supported by thermal shift assays, reveal that these mutations significantly destabilize the protein structure. Pharmacological interventions with proteasome inhibitors MG-132 and bortezomib moderately rescue the functional deficits of R44Q, while treatment with trichostatin A, enhances both *in vivo* expression and *in vitro* activity of this variant. Utilizing a *Drosophila* model system - where GAT expression is restricted to astrocytes alongside the glutamate transporter - we observed a rapid, proteasome-dependent degradation of R44Q. On the other hand, in GABAergic neurons, R44Q exhibited diminished expression and notable presynaptic localization. R44Q showed a distinctive cell-type specific expression pattern, compared to other, previously reported, SLC6 transporter mutants, which generally display consistent phenotypes across cell types. Interestingly, the R44Q variant - unresponsive to 4-PBA treatment *in vitro* - exhibited elevated astrocytic expression and marked amelioration of seizure-like phenotypes upon treatment *in vivo*. Together, our findings reveal mechanistic insights into restoring function in disease-associated SLC6 missense variants and suggest broader strategies for correcting pathogenic mutations across this transporter family.

## Data Availability

The raw data supporting the conclusions of this article will be made available by the authors, without undue reservation.

## Supplementary material

SUPPLEMENTARY FIGURE S1 Quantification of mature band intensities of WT hGAT-1 and the R44Q mutant upon trichostatin A treatment. The mature glycosylated bands (“M” bands) were quantified and the intensities normalized to Gβ were plotted relative to their own untreated control condition. The data were obtained from three independent experiments (error bars=SEM). The normalized values were statistically compared by one-way ANOVA, followed by Tukey’s post hoc t-tests and showed no significant changes among the tested conditions (* p > 0.05).
