# Supplementary figures and images for: Pathogenic variants at the N-terminal arginine residue 44 disrupt human GABA transporter 1 function: insights from Drosophila epilepsy models
Source: Front Pharmacol. 2025 Nov 25;16:1674737. doi: 10.3389/fphar.2025.1674737 (PMC12685850; doi:10.3389/fphar.2025.1674737)

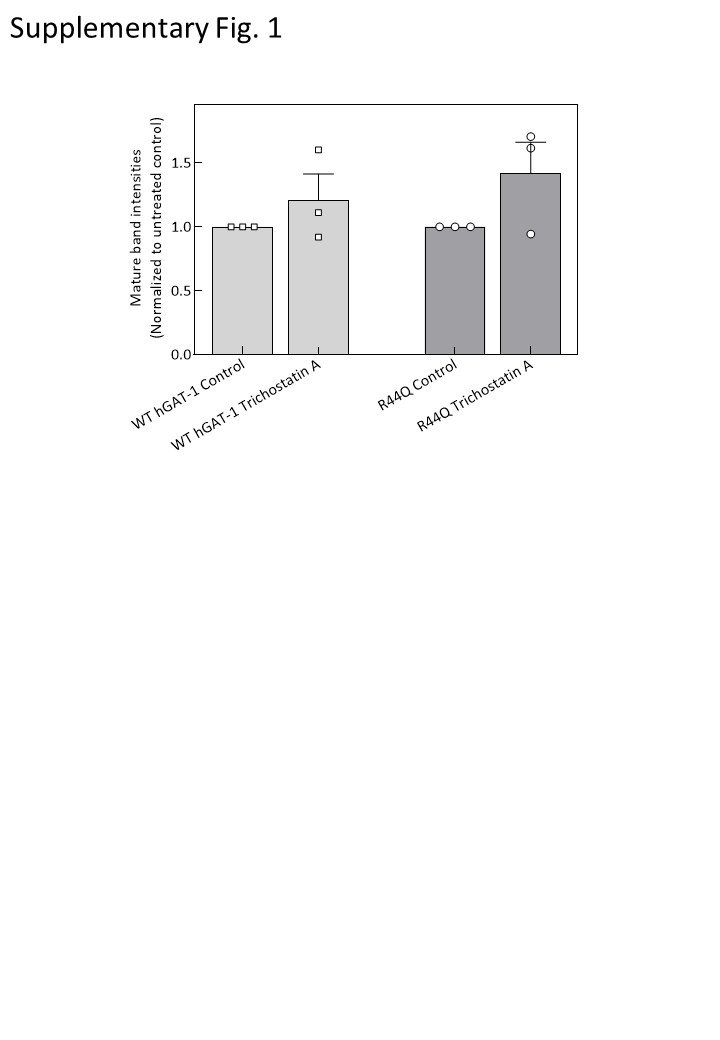

Supplement: Supplementary file 1 [file Image1.tif]
